# Teaching Conflicts of Interest and Shared Decision-Making to Improve Risk Communication: a Randomized Controlled Trial

**DOI:** 10.1007/s11606-019-05420-w

**Published:** 2019-12-10

**Authors:** Cora Koch, Nadine Dreimüller, Janosch Weißkircher, Nicole Deis, Eva Gaitzsch, Stefanie Wagner, Marlene Stoll, Franziska Bäßler, Klaus Lieb, Jana Jünger

**Affiliations:** 1 Department of Psychiatry and Psychotherapy, University Medical Center Mainz, Mainz, Germany; 2 Department of Neurology and Neurophysiology, University Medical Center Freiburg, Freiburg, Germany; 3 Department of Pneumology, Thoraxklinik Heidelberg, Heidelberg University Hospital, Heidelberg, Germany; 4 Department for General Internal and Psychosomatic Medicine, Heidelberg University Hospital, Heidelberg, Germany; 5 IMPP - German Institute for Medical and Pharmaceutical Examinations, Postfach 2528, 55015 Mainz, Germany

**Keywords:** Shared decision-making, Risk communication, Conflict of interest, Medical education

## Abstract

**Background:**

Risk communication is a core aspect of a physician’s work and a fundamental prerequisite for successful shared decision-making. However, many physicians are not able to adequately communicate risks to patients due to a lack of understanding of statistics as well as inadequate management of conflicts of interest (COI).

**Objective:**

To evaluate the effects of an integrated curriculum encompassing COI and shared decision-making on the participants’ risk communication competence, that is, their competence to advise patients on the benefits and harms of diagnostic or therapeutic interventions.

**Design:**

A rater-blind randomized controlled trial with a 30 (± 1)-week follow-up conducted from October 2016 to June 2017 at two German academic medical centers.

**Participants:**

Sixty-three medical students in their fourth or fifth year.

**Interventions:**

Participants received either a newly developed 15-h curriculum or a course manual adapted from teaching as usual.

**Main Measures:**

Primary outcome: change in risk communication performance in a video-observed structured clinical examination (VOSCE).

**Key Results:**

Participants were 25.7 years old on average (SD 3.6); 73% (46/63) were female. Increase in risk communication performance was significantly higher in the intervention group with post-intervention Cohen’s *d* of 2.35 (95% confidence interval (CI) 1.62 to 3.01, *p* < 0.01) and of 1.83 (CI 1.13 to 2.47, *p* < 0.01) 30 (± 1) weeks later. Secondary outcomes with the exception of frequency of interactions with the pharmaceutical industry also showed relevant improvements in the intervention as compared with the control group (*d* between 0.91 and 2.04 (*p* < 0.001)).

**Conclusions:**

Our results show that an integrated curriculum encompassing COI and risk communication leads to a large and sustainable increase in risk communication performance. We interpret the large effect sizes to be a result of the integration of topics that are usually taught separately, leading to a more effective organization of knowledge.

**Trial Registration:** The trial is registered in the International Clinical Trials Registry with the trial number DRKS00010890.

**Electronic supplementary material:**

The online version of this article (10.1007/s11606-019-05420-w) contains supplementary material, which is available to authorized users.

## INTRODUCTION

Risk communication is a core aspect of a physician’s work and a fundamental prerequisite for successful shared decision-making.^1–3^ However, risk communication is difficult because it requires not only a precise understanding of the harms and benefits of an intervention—whether diagnostic or therapeutic—but also the communicative skills to communicate these risks in a way that is understandable for patients. Studies show that many physicians are not up to the challenge.^4–8^

For one, studies suggest that many physicians cannot accurately interpret statistics commonly used to describe effects of screening and therapeutic interventions; some have called this “statistical illiteracy.”^5, 7^ For example, many physicians rely on the wrong statistical parameters to judge the effect of screenings for common cancers^6^ and are not able to explain basic statistic concepts such as absolute or relative risk reduction.^7^ Conflicts of interest (COI) resulting from interactions with the pharmaceutical industry further impede the interpretation of risks: they lead to a biased view on risk information as well as a biased presentation of scientific data in information brochures and scientific publications.^9–11^ Physicians thus need to avoid such conflicts or manage them professionally to avoid bias. Yet data from the Physician Payments Sunshine Act as well as surveys among physicians show that interactions with pharmaceutical companies continue to be pervasive.^12–14^

According to our literature search and knowledge from a curricular mapping project, most aspects of risk communication are being taught in medical school—however, they are taught separately.^15^ Some curricula on the topics have been published,^16–18^ but no integrated curriculum encompassing all aspects important for risk communication exists in the literature. We thus hypothesize that an integration of the relevant topics—basic statistics, bias detection, professional management of COI, and communication skills—is necessary to improve physicians’ risk communication competence by improving knowledge organization, facilitating knowledge transfer into practice, and emphasizing the importance of basic statistics and professionalism for the treatment quality of patients. We developed an integrated curriculum covering these topics and choosing didactic methods primarily based on adult learning theory.^19^ The objective of this study was to evaluate the effects of this curriculum on the participants’ risk communication performance.

## METHOD

### Ethics Review

This study received a positive ethics review from the two responsible ethics committees for Mainz and Heidelberg (Ethik-Kommission der Ärztekammer Rheinland-Pfalz, case number 837.053.16 (10372); and Ethikkommission der Medizinischen Fakultät Heidelberg, case number S-314/2016)

### Design and Setting

This was a rater-blind randomized controlled trial conducted from October 2016 to June 2017 at two German academic medical centers (Mainz and Heidelberg). Eligible for participation were all medical students in their fourth and fifth year at the two universities. All participants had had training in statistics and basic communication skills. Each student who completed the entire study received a cash incentive of 100 €.

### Sample Size

It was calculated that a sample size of 60 participants (30 per group) was necessary to guarantee a power of 0.85 to detect an effect size of 0.8 standard deviations regarding the primary outcome (risk communication performance as measured in a video-observed structured clinical examination (VOSCE), see below).

### Procedure

Participating students provided informed consent before completing a baseline assessment consisting of a VOSCE as well as a questionnaire (details see below). The students were then randomized 1:1 to the intervention or control group. After receiving the respective intervention, participants completed a similar assessment twice more: once up to 2 weeks after the intervention (post-test) and once 30 (± 1) weeks later (follow-up).

### Randomization and Blinding

Randomization to the two groups was stratified by knowledge (see below, “Secondary outcome measures”) at baseline.^20^ The randomization sequence was generated by the IMBEI in Mainz. Participants could not be blinded to the allocation because of the nature of the intervention. However, raters and standardized patients (SP) were blinded to the allocation of the participants.

### Interventions

The intervention group received an integrated curriculum developed based on a literature review and expert opinions (see online Appendix Table 1 for an overview of curriculum content and methods). The process for the development of the curriculum was based on the six-step approach described by Kern et al.^21^ For the choice of methods, we used principles of adult learning theory and integrated activities in the active mode of learning according to the “ICAP hypothesis” and used the concept of deliberate practice to design practice sessions.^19, 22, 23^

A first version of the curriculum was piloted with 13 medical students in Heidelberg. The curriculum was then adjusted according to feedback from the participants. The final curriculum covered the most essential statistic concepts to judge the efficacy and risks of treatment options and screenings, theoretical and practical skills in risk communication, and an overview of relevant COI in health care such as sponsorship of research by industry or acceptance of personal gifts, how they may bias the interpretation of risk information, and how COI can be managed to avoid bias (see online Appendix Table 1 for more detail). In all, the course was taught three times in November and December of 2016. The control group received a course manual that was adapted from existing lecture notes from separately taught mandatory courses on communication, statistics, and epidemiology at the Universities of Heidelberg and Mainz (“teaching as usual”); it did not contain information on conflicts of interest, as these are currently not explicitly taught. Participants in both control and intervention groups had participated in the courses prior to participation in our study. Participants in the control group were instructed to revisit the material using our adapted course manual before participating in the post-test (Summary of course manual in online Appendix Table 2).

### Primary Outcome Measure

The primary outcome measure was the change in risk communication performance of the students, measured in a VOSCE.^24^ It consisted of two consultations in which the students were asked to communicate to an SP the risks regarding a therapeutic or screening intervention, respectively (see online Appendix 1 for further information). An hour prior to the consultation, students were given informational material on the respective intervention that showed a bias toward the intervention. The consultations were video-taped and rated using an adapted version of the rating scale developed by Han et al.,^18^ which consists of 20 items rating risk communication process (RCP), i.e., generic communication skills, and risk communication content (RCC), i.e., the key risk information being communicated. Students could score a maximum possible of 120 points for the two consultations, 72 for risk communication process, and 48 for risk communication content (see online Appendix Table 3 for a list of items on the scale and online Appendix 2 for more detail on the process of adapting the scale as well as scoring details).

Each video was rated independently by two raters. Raters were trained to use the scale by ND and CK. Inter-rater reliability for the rating of the videos was good to excellent with an ICC of 0.884 (CI 0.794 to 0.935). For the final analysis, means of the two ratings were used.

### Secondary Outcome Measures

Secondary outcomes were changes in knowledge, attitudes, professionalism regarding COI, frequency of interactions with the pharmaceutical industry, and self-assessed interest, knowledge, and competence.

Knowledge regarding risk communication and COI was assessed in a 30-item multiple choice questionnaire. Attitudes regarding COI were assessed in a 10-item questionnaire adapted from Sierles et al. for the situation in Germany,^25, 26^ from which a skepticism score was calculated where higher scores indicate higher skepticism.^25^ Professionalism regarding the management of COI was assessed using a situational judgment test (SJT) encompassing five scenarios involving a COI and five possible ways to behave in each. Students could score a maximum of 125 points on the SJT (see online Appendices 3 and 4 for an example of SJT and details of scoring it). Frequency of interactions with the pharmaceutical industry was assessed using a 12-item questionnaire regarding six different types of interactions where students were asked to name the number of times they had been offered or had engaged in this interaction within the last 6 months. Self-assessed interest, knowledge, and competence were assessed using an 11-item questionnaire that was adapted from Brown et al.^27^

### Data Analysis

Data were analyzed using SPSS 23. To test for group differences between the control and intervention groups regarding continuous outcome measures, two-tailed *t* tests for independent variables were used and Cohen’s *d* calculated. Effect sizes were only calculated for those participants that participated in the respective assessments (post-test/follow-up). A *p* < 0.05 was regarded to be significant for the primary outcome. Results are reported as Cohen’s *d* with 95% confidence interval. The McNemar test was used to test for a bias blind spot. To assess the success of the rater training and the inter-rater reliability for the VOSCE within the study, the average measures, absolute agreement, and two-way random effects ICC were calculated.

## RESULTS

### Participants

**Figure 1:**
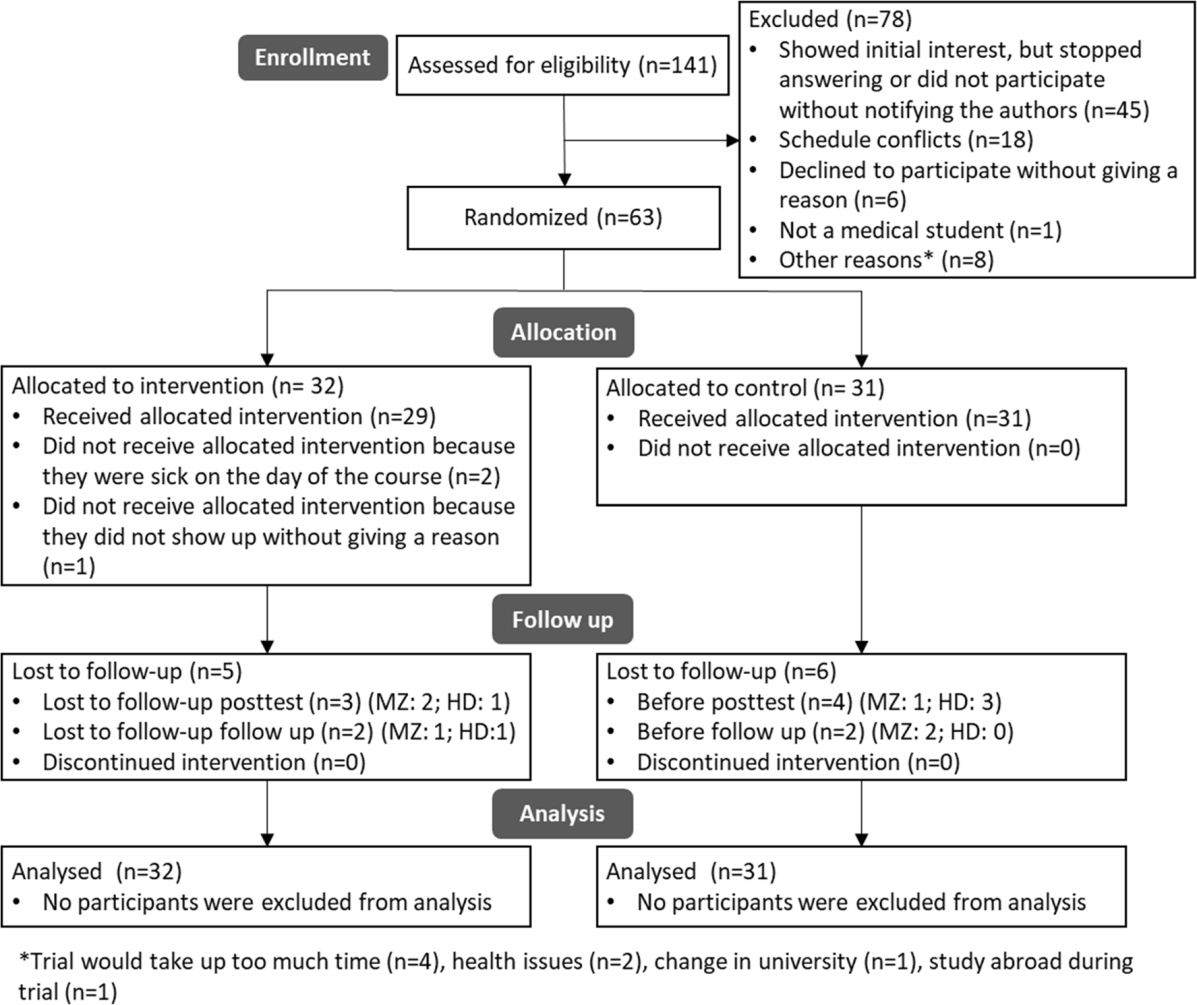
Recruitment of participants.

Figure 1 details the recruitment of participants. Sixty-three participants were randomized; they had a mean age of 25.7 (SD 3.6), 46/63 (73%) were female, and on average, they were in their 9th semester of study (8.8; SD 1.3). In the control group, participants also had a mean age of 25.7 (SD 3.7), 25/32 (78%) were female, and they were in their 9th semester of study (8.8; SD 1.1). In the intervention group, participants had a mean age of 25.6 (SD 3.6), 21/31 (68%) were female, and they were in their 9th semester of study (8.9; SD 1.5) (see Tables 1 and 2 for baseline results for primary and secondary outcomes).]-->

**Table 1:** Risk Communication Performance

Parameter	Baseline	Post-test	Δ (post-test)	Follow-up	Δ (follow-up)
Risk communication performance					
Intervention group mean (SD)	48.36 (7.51)	70.07 (11.8)	21.71 (10.24)	68.46 (10.67)	19.96 (10.46)
Control group mean (SD)	49.65 (8.12)	50.74 (7.23)	0.98 (6.81)	52.26 (5.59)	3.14 (7.12)
Difference between intervention and control (95% CI; *p*)	-	-	20.73 (15.9 to 25.56; < 0.001)	-	16.82 (11.49 to 22.16; < 0.001)
Cohen’s *d* (95% CI)	-	-	2.35 (1.62 to 3.01)	-	1.83 (1.13 to 2.47)
Risk communication process					
Intervention group mean (SD)	44.76 (5.49)	49.34 (5.70)	4.59 (5.83)	50.39 (4.13)	5.43 (6.91)
Control group mean (SD)	44.71 (6.77)	45.26 (5.74)	0.44 (5.41)	46.31 (3.98)	1.45 (5.03)
Difference between intervention and control means (95% CI; *p*)	-	-	4.15 (1.06 to 7.24; 0.01)	-	3.98 (0.39 to 7.57; 0.03)
Cohen’s *d* (95% CI)	-	-	0.74 (0.17 to 1.28)	-	0.64 (0.05 to 1.21)
Risk communication content					
Intervention group mean (SD)	3.60 (2.05)	20.73 (8.44)	17.12 (7.70)	18.07 (9.10)	14.54 (8.34)
Control group mean (SD)	4.94 (4.54)	5.48 (4.20)	0.54 (3.27)	5.95 (3.38)	1.69 (4.33)
Difference between intervention and control means (95% CI; *p*)	-	-	16.58 (13.25 to 19.91; < 0.001)	-	12.85 (8.83 to 16.86; < 0.001)
Cohen’s *d* (95% CI)	-	-	2.73 (1.95 to 3.43)	-	1.86 (1.14 to 2.50)

Δ (post-test), difference between post-test and baseline; Δ (follow-up), difference between follow-up and baseline; intervention group *n* at baseline 29, post-test 29, follow-up 28; control group *n* at baseline 26, post-test 25, follow-up 21

**Table 2:** Secondary Outcomes—Overview

	Baseline	Post-test	Δ (post-test)	Follow-up	Δ (follow-up)
Knowledge (multiple choice questionnaire)					
Intervention group mean (SD)	16.63 (3.30)	24.00 (3.92)	7.52 (3.04)	20.93 (3.51)	4.46 (2.33)
Control group mean (SD)	16.35 (4.29)	17.93 (3.62)	1.96 (2.33)	18.38 (4.15)	2.08 (2.92)*
Difference intervention and control means (95% CI; *p*)	-	-	5.55 (4.09–7.01; < 0.001)	-	2.38 (0.92–3.84; < 0.001)
Cohen’s *d* (95% CI)	-	-	2.04 (1.37–2.65)	-	0.91 (0.32–1.47)
Skepticism (attitude questionnaire)					
Intervention group mean (SD)	0.59 (0.17)	0.76 (0.14)	0.19 (0.17)	0.76 (0.17)	0.19 (0.20)^†^
Control group mean (SD)	0.51 (0.15)	0.54 (0.14)	0.03 (0.10)	0.54 (0.17)	0.02 (0.14)
Difference intervention and control means (95% CI; *p*)	-	-	0.16 (0.08–0.23; < 0.001)	-	0.17 (0.07–0.27; < 0.001)
Cohen’s *d* (95% CI)	-	-	1.15 (0.57–1.69)	-	0.96 (0.38–1.52)
Professionalism regarding COI (situational judgment test)					
Intervention group mean (SD)	66.22 (14.02)	84.48 (16.00)	18.66 (12.06)	85.28 (17.11)	19.12 (15.75)^‡^
Control group mean (SD)	55.61 (15.06)	55.96 (16.55)	0.27 (7.75)	60.04 (21.76)	3.35 (12.94)^§^
Difference intervention and control means (95% CI; *p*)	-	-	18.39 (12.83–23.94; < 0.001)	-	15.77 (7.36–24.19; < 0.001)
Cohen’s d (95% CI)	-	-	1.79 (1.14–2.39)	-	1.09 (0.47–1.68)

Δ (post-test), difference between post-test and baseline; Δ (follow-up), difference between follow-up and baseline; intervention group *n* at baseline 32, post-test 29, follow-up 28; control group *n* at baseline 31, post-test 27, follow-up 25; *n = 24, ^†^*n* = 27, ^‡^*n* = 25, ^§^*n* = 23

### Primary Outcome

**Figure 2:**
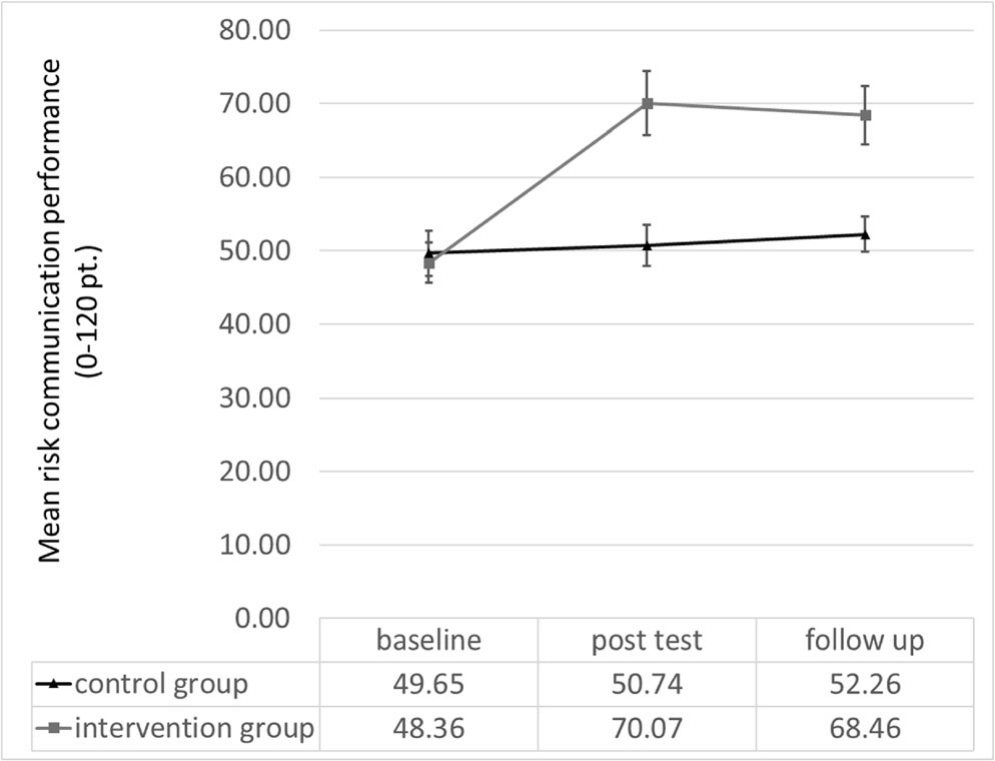
Risk communication performance (error bars denote standard deviation).

Table 1 gives an overview of the results of risk communication performance. The change in risk communication performance compared with that in baseline was significantly larger in the intervention group for both the post-test and the follow-up with a Cohen’s *d* of 2.35 (CI 1.62 to 3.01, *p* < 0.01) and 1.83 (CI 1.13 to 2.47, *p* < 0.01), respectively. The difference between the two groups thus persisted until follow-up, though it narrowed slightly (see Fig. 2 for a graphic representation). In Germany, a criterion-based passing score is usually set at 60% of the full score. Assuming this as a passing score, 42.9% (12/28) of the intervention group would have passed the VOSCE at post-test and 40.7% (11/27) at the follow-up, while none of the control group participants would have passed at either time point.]-->

### Secondary Outcomes

Table 2 gives an overview of the secondary outcomes regarding knowledge, attitudes, and professionalism regarding COI. Data from the self-assessment questionnaire is presented in online Appendix Table 4.

### Knowledge

Increase in knowledge was larger in the intervention than in the control group (*d* = 2.04 (CI 1.37 to 2.65; *p* < 0.01) at post-test and 0.91 (CI 0.32 to 1.47; *p* < 0.01) at follow-up). Assuming the abovementioned passing score of 60%, at post-test, 27/29 (93.1%) of the intervention group and 16/27 (59.3%) of the control group would have passed. At follow-up, 23/28 (82.1%) of the intervention group and 17/24 (70.8%) of the control group would have passed.

### Attitudes

Skepticism increase in the intervention group was larger than in the control group (*d* = 1.15 (CI 0.57 to 1.69; *p* < 0.01) at post-test and 0.96 (CI 0.38 to 1.52; *p* < 0.01) at follow-up). Looking at the items individually, the intervention group showed a trend toward more skeptical attitudes that persisted until follow-up in all attitude items. Table 3 shows individual item data for the most relevant attitude items regarding bias detection in risk information.

**Table 3:** Relevant Individual Item Data for Attitudes on COI (on a Likert Scale from 0 to 3; Scaled so that a higher score signals a more skeptical attitude)

Item		Baseline	Post	Δ post		Follow-up	Δ follow-up	
		*M* (SD)	*M* (SD)	*M* (SD)	*p*	*M* (SD)	*M* (SD)	*p*
Materials from a pharmaceutical company are helpful to inform oneself about new drugs.	Int	1.63 (0.71)	2.38 (0.56)	0.79 (0.78)	< 0.01	2.26 (0.71)	0.70 (0.87)	< 0.01
	Con	1.52 (0.72)	1.33 (0.55)	− 0.26 (0.90)	0.15	1.20 (0.71)	− 0.48 (0.77)	< 0.01
	Diff. between Int and Con	-	-	1.05	< 0.01	-	1.18	< 0.01
Most CME events or grand rounds sponsored by pharmaceutical companies are helpful and informative.	Int	1.75 (0.67)	2.17 (0.60)	0.45 (0.74)	< 0.01	1.96 (0.65)	0.30 (0.82)	0.073
	Con	1.19 (0.60)	1.30 (0.47)	0.15 (0.66)	0.26	1.44 (0.77)	0.28 (0.79)	0.09
	Diff. between Int and Con	-	-	0.3	0.12	-	0.016	0.94
CME events or grand rounds sponsored by pharmaceutical companies are usually biased in favor of the company’s product.	Int	2.56 (0.50)	2.83 (0.38)	0.24 (0.69)	0.07	2.65 (0.75)	0.08 (0.80)	0.63
	Con	2.42 (0.67)	2.56 (0.51)	0.15 (0.60)	0.21	2.48 (0.59)	0.08 (0.70)	0.57
	Diff. between Int and Con	-	-	0.093	0.59	-	0	0.99
It is sometimes acceptable for medical students to accept gifts or lunch from pharmaceutical companies because pharmaceutical companies have minimal influence on students.	Int	2.09 (0.86)	2.45 (0.74)	0.41 (0.82)	0.01	2.56 (0.58)	0.59 (0.84)	< 0.01
	Con	1.81 (1.08)	1.74 (0.90)	− 0.11 (0.97)	0.56	1.92 (0.78)	0.08 (1.01)	0.60
	Diff. between Int and Con	-	-	0.52	0.03	-	0.51	0.057

*Int* intervention group, *Con* control group, Δ change within group from baseline

At baseline, more participants in both the intervention and the control groups thought that others were more likely to be influenced by gifts from pharmaceutical sales representatives than themselves, which has been described as evidence for a “bias blind spot.”^28^ At post-test and follow-up, this difference was not seen in the intervention group (see Table 4), while it remained in the control group.

**Table 4:** Bias Blind Spot

	Baseline	Post-test	Follow-up
Intervention group, *n*	32	29	26
Influence on me*	14 (43.8%)	23 (79.3%)	24 (92.3%)
Influence on others^†^	21 (65.6%)	25 (86.2%)	24 (92.3%)
*p*	0.039	0.5	1
Control group, *n*	31	27	25
Influence on me*	12 (38.7%)	14 (51.9%)	14 (56.0%)
Influence on others^†^	21 (67.7%)	20 (74.1%)	19 (760%)
*p*	0.004	0.031	0.13

*Agreement with the statement “Accepting gifts or food from a drug rep increases the likelihood that I will later prescribe the drugs of that company”

†Agreement with the statement “Accepting gifts or food from a drug rep increases the likelihood that my fellow students will later prescribe the drugs of that company”

### Professionalism Regarding COI

The improvement in professionalism regarding COI was higher in the intervention group at post-test as well as follow-up with *d* = 1.79 (CI 1.14 to 2.39, *p* < 0.01) and 1.09 (CI 0.47 to 1.68, *p* < 0.01), respectively. These results were similarly found for each scenario separately (see online Appendix Table 5).

### Acceptance of Gifts

At baseline, 21% (13/62) of all participants indicated that they had not interacted with pharmaceutical companies within the last 6 months. This percentage increased to 39.2% (20/51) at follow-up; however, there was no relevant difference between the intervention and control groups (intervention group 40.7% (11/27), control group 37.5% (9/24)). Further data on gift acceptance can be found in online Appendix Table 6.

## Discussion

The integrated curriculum on conflicts of interest and risk communication we developed led to a large, statistically significant increase in risk communication performance of the participants compared with the control group at both post-test and follow-up 30 weeks later. In addition, the intervention group showed increased knowledge regarding both risk communication and COI and a change in attitudes toward more skepticism toward interactions with pharmaceutical companies as well as improved intentions of managing COI in a way that reduces their risk of bias. However, there was no change in acceptance of gifts or attendance of sponsored events. At follow-up, differences between the two groups decreased slightly for all measures. This was especially pronounced for the MC knowledge exam, where the intervention group score declined while the control group score increased slightly. Risk communication performance and attitude items, however, were relatively stable.

The change in risk communication competence is generally in line with the results of one other study evaluating a curriculum on the topic. In this single blind controlled trial, the intervention group also had significantly better scores for objective risk communication competence (content as well as process) than the control group.^18^ Regarding the baseline skepticism, students in our study were comparable with previous German and international studies, albeit slightly more skeptical.^25, 26^ In studies on curricula regarding COI, similar changes in attitude were found as in this study. However, none of these had a comparable follow-up and effects seen were usually somewhat smaller or less consistent than in this study.^16^ To our knowledge, no study has assessed the management of COI by students using an SJT. Previous studies assessing the effect of curricula on accepting gifts have had contradictory results.^16^

We suspect that the large effects we found are due to the integration of topics that are usually taught separately. This is supported by the fact that all the students had had courses on almost all of the topics covered in our curriculum and still showed a large learning gain. In our view, this cannot be exclusively due to the coverage of novel content, because only very little content, mostly pertaining to conflicts of interest, was taught to the intervention but not the control group. One of the reasons why integrated curricula may lead to a larger learning effect is that they support a knowledge organization by students that is better matched to the tasks that are required of them.^29^ When topics are taught separately, students organize knowledge around separate categories and may fail to make connections for tasks that require incorporating different areas of expertise. Teaching the topics together with a clear structure denoting how the topics are relevant to the tasks may thus lead to a larger learning gain. Additionally, following adult learning theory, the practical exercises based on situations encountered during clinical practice underscore the relevance of topics to the participants’ daily lives, leading to a larger motivation to learn.^19^ Despite the large learning gains, only 40% of students in the intervention group would have passed the VOSCE when applying a criterion-based passing score of 60%. We think this may be due to a combination of factors: our rating scale is not externally validated, so it is unclear which score corresponds to a sufficient mastering of the task; students may not have prepared for the formative assessment within our study as intensively as they would have for a grade-relevant exam; and finally, the students may have needed more time to master this highly complex task.

In addition, the gain in risk communication competence was enduring over the 30-week follow-up, unlike the knowledge gain. Possibly, risk communication is a form of procedural knowledge less prone to decline. In addition, the intense (“deliberate”) practice of this skill during the curriculum using both role play and training with SPs could explain that this competence was more enduring than the declarative knowledge needed for the MC exam.^23^

It is unclear why the marked change in attitudes as well as behavioral intent did not translate to a change in behavior regarding accepted gifts or attended sponsored events. This could be due to environmental factors, conforming with the social cognitive theory of behavior.^30^ Furthermore, the prediction of behavior from attitudes is generally difficult.^31, 32^ Lastly, our study was only powered to detect a difference in the primary outcome, possibly explaining why we did not find a difference regarding gift acceptance.

This study had several strengths. One was the meticulous development process for the integrated curriculum, which resulted in a well-crafted course with a high acceptance by the participants. Methodically, the randomized controlled design is a major strength of this study. It is the first study to investigate the effects of a curriculum on COI or risk communication with such a design.^16^ The long follow-up period is also an advantage compared with other trials of curricula covering similar topics. A weakness of our study is that the evaluation instruments were not formally validated. However, they had been piloted and the rating scale for the VOSCE showed a good internal consistency and high inter-rater reliability.

The fact that most of the individual topics necessary for risk communication had already been taught to participants previously supports our hypothesis that the large learning gains we found are mostly due to our method of integration of topics rather than due to the teaching of previously unknown subject matter. Consequently, an entrustable professional activity testing clinical decision-making under the consideration of COI has been introduced as a requirement for the licensing examinations in Germany following the results of this study. However, our study was not designed to test which elements of the curriculum were responsible for the large effects. Further studies are needed to identify whether our hypothesis that integration is the reason for the large effects holds true and to demonstrate the implementation at other universities is feasible and leads to comparably strong effects as seen in our study.

## Electronic Supplementary Material

ESM 1 (DOCX 41.5 kb)

## Data Availability

The data sets analyzed during this study are available from the corresponding author upon reasonable request.
