# Supplementary material for: Teaching Conflicts of Interest and Shared Decision-Making to Improve Risk Communication: a Randomized Controlled Trial
Source: J Gen Intern Med. 2019 Dec 10;35(2):473–80. doi: 10.1007/s11606-019-05420-w (PMC7018798; doi:10.1007/s11606-019-05420-w)
Supplement: Supplementary file 1 — (DOCX 41.5 kb) [file 11606_2019_5420_MOESM1_ESM.docx]

**MS #27531**

**Teaching Conflicts of Interest and Shared Decision Making to improve Risk Communication: a Randomized Controlled Trial**

**Appendix**

*Appendix Table 1. Overview of the integrated curriculum with time (in min) allocated to each topic*

| **Content** | **Method** | **Time** |
| --- | --- | --- |
| **Day 1** | | |
| **Introduction to Risk Communication** | Interactive Presentation | 30 |
| Definition of risk |  |  |
| Characteristics of risk information |  |  |
| Importance of risk communication |  |  |
| **Introduction to Conflicts of Interest** | Interactive Presentation | 45 |
| Definition of COI |  |  |
| Types of COI in the context of health care |  |  |
| Frequency of interactions with pharmaceutical industry |  |  |
| Effects of COI |  |  |
| **Basic Statistics – Screening** | Presentation broken up by exercises students work on individually or in pairs | 60 |
| Survival vs. mortality rate; leadtime and overdiagnosis bias | Discussion in pairs: Recommendation of a prostate cancer screening based on survival vs. based on mortality rate |  |
| Relative vs. absolute risk reduction; number needed to treat | Exercise: Calculation of RRR, ARR and NNT starting with different base rates and comparison of the results |  |
| Conditional probability vs. natural frequencies |  |  |
| Positive predictive value | Exercise: Calculation of probability that a patient has colon cancer from a positive stool test |  |
| **Patient Decision Aid** | Short presentation | 60 |
| Use of fact boxes as a decision aid for patients | group work: participants create a fact box regarding an ovarian cancer screening using data from a systematic review |  |
| **Day 2** |  |  |
| **Basic Statistics - Treatment** | Interactive presentation | 90 |
| p-values | after input, students are asked to interpret p-values and confidence intervals regarding statistic significance and clinical relevance |  |
| Confidence intervals |  |  |
| Kaplan-Meier curves | after input, students are asked to interpret an example |  |
| Introduction into Cochrane reviews: forrest plots, meta analyses |  |  |
| **Psychological Aspects of Conflicts of Interest** | Short input presentation followed by group work and discussion | 45 |
| Definition of cognitive bias esp. self-serving bias  Psychology of influence: reciprocity, consistency, social desirability, sympathy and authority | Group work in pairs on two case vignettes (a pediatrician accepting samples and participating in a post-marketing observational study; a pulmological fellow doing research in cooperation with pharmaceutical companies and presenting in CME-events)  Group discussion with all students on psychological mechanisms at work in the vignettes |  |
| **Presentation of scientific data** | Interactive presentation and group work | 75 |
| Sources for bias in trial design  Publication bias  Spin  Biased presentation of results (absolute vs. relative risks, distorted graphs, etc.) | Analysis of a graph in the group  Group discussion comparing two different texts on nalmefene (a pharma brochure and an independent text) |  |
| **Risk communication and shared decision making** | short input presentation followed by role play | 120 |
| Shared decision making: definition, importance, opportunities for use |  |  |
| Risk communication: definition and process (giving an overview, exploring the patients’ preferences, imparting risk information, summarizing the essential information, verifying patient understanding and eliciting questions) | Role Play: Students recreate a consultation with one student as doctor, one as patient and one giving feedback. Each student gets the chance to play each role. Cases: breast cancer prevention, home birth and stroke prevention |  |
| **Minimizing Bias Resulting from Conflicts of Interest** | Presentation and group discussion | 45 |
| COI as misconduct and relevant guidelines/laws  Principles for reducing influence of COI | Group discussion of an example: qualification of a doctor with conflicts of interests to participate in a clinical guideline committee |  |
| Avoidable COI |  |  |
| Independent informational materials |  |  |
| **Day 3** | | |
| **Practice of risk communication competence** |  |  |
| Introduction into the SP-sessions | Short input presentation | 30 |
| Individual preparation for SP sessions | Students prepare individually using the patient vignettes and informational material on one of the four respective cases (medication to prevent relapse in alcohol abuse, operation in the treatment of a disc prolapse, psa screening and chemotherapy for oesophageal cancer) | 60 |
| Group preparation for SP sessions | Group puzzle: students introduce their own case to other students that worked on a different case and discuss | 60 |
| Four consultations with SPs including feedback (one consultation per student) | Students consult with SPs regarding a screening or therapeutic intervention; afterward, the student, the SP, the teacher as well as one of the other students give feedback. | 120 |
| Questions and Feedback | Students can ask questions that have remained unanswered as well as give feedback on the course. | 30 |

*Appendix Table 2. Summary of Course manual that the control group received*

| Basic statistics |
| --- |
| Percent, Risk and Rate |
| Screening |
| Confidence Intervals |
| Statistic Tests and p-value |
| Glossary |
| Physician-Patient Communication |
| Tips for the communication between doctor and Patient |
| Taking a history in an urgent care setting |
| Models for consultations |
| Shared decision making (SDM) |
| Phases of a conversation according to the SDM-Model |
| Guideline for a structured consultation |
| Guideline for conflict- and feedback consulations (esp. CALM-model) |
| Guideline for informed consent |
| Guideline for delivering bad news (SPIKES-Model) |
| Guideline for communication with deaf patients |

*Appendix Table 3. Rating scale for risk communication performance, adapted following Han et al. (see [12])*

| Risk communication process (RCP) |
| --- |
| 1. Greeted patient appropriately and introduced self |
| 2. Set the stage for discussion |
| 3. Assessed preferences for information |
| 4. Checked understanding, elicited questions |
| 5. Gave information clearly using plain language, avoided jargon |
| 6. Elicited patient concern and responded appropriately |
| 7. Gave a well-paced explanation |
| 8. Maintained a dialogue |
| 9. Demonstrated empathy |
| 10. Actively listened |
| 11. Built a therapeutic relationship throughout the consultation |
| 12. A decision was made (incl. to defer the decision, where appropriate) |
| Risk communication content (RCC) |
| 1. Discussed the quality/strength/weakness (e.g., validity, reliability, credibility) of the risk evidence |
| 2. Specified the reference class (patient population) for whom the risk estimates apply |
| 3. Specified the time period over which the risk estimates apply |
| 4. Explained the magnitude of risk using both negative and positive frames |
| 5. Explained risk estimates using proportions (e.g., ‘‘9 out of 100’’) |
| 6. Discussed differences between baseline risk and modified risk in absolute terms (absolute risk reduction) or both absolute and relative terms |
| 7. Acknowledged general uncertainty in all risk estimates |
| 8. Acknowledged uncertainty due to chance or randomness (inability to predict single events) |

*Appendix Table 4. Self-assessment scores at baseline, posttest and follow up*

| **Statement** | **Baseline** |  | **Posttest** |  | **Follow up** |  |
| --- | --- | --- | --- | --- | --- | --- |
|  | Int | Con | Int | Con | Int | Con |
| n | 32 | 31 | 29 | 27 | 27 | 25 |
| I am interested in conflicts of interest. | 5.1 (0.8) | 4.8 (0.9) | 5.1 (0.7) | 4.8 (0.9) | 5.0 (0.7) | 4.7 (0.9) |
| I am aware of frequent types of conflicts of interest. | 4.3 (0.9) | 4.1 (0.9) | 4.8 (0.9) | 4.1 (1.0) | 4.8 (0.6) | 4.1 (0.8) |
| I know sources of neutral information. | 4.1 (1.3) | 4.1 (1.3) | 5.1 (0.7) | 4.0 (1.3) | 5.0 (0.9) | 4.3 (1.2) |
| I know the psychological mechanisms of conflicts of interest. | 3.6 (1.1) | 3.3 (1.1) | 5.0 (0.7) | 3.4 (1.3) | 4.7 (0.8) | 3.8 (1.1) |
| I am interested in risk communication. | 5.2 (0.8) | 5.1 (0.7) | 5.0 (0.8) | 4.9 (1.0) | 4.9 (0.9) | 5.0 (0.8) |
| I feel secure handling medical statistic data. | 2.5 (1.2) | 2.7 (1.1) | 4.2 (0.7) | 2.7 (1.3) | 3.6 (1.0) | 3.0 (1.2) |
| I know important elements of risk communication. | 2.6 (0.9) | 2.8 (1.0) | 4.9 (0.7) | 3.0 (1.1) | 4.1 (0.7) | 3.0 (1.1) |
| I can communicate medical statistics to patients well. | 2.7 (1.0) | 3.2 (1.0) | 4.3 (0.6) | 3.0 (1.1) | 3.9 (0.5) | 3.2 (1.0) |
| I can communicate probabilities in an understandable way. | 3.0 (1.0) | 3.5 (1.0) | 4.5 (0.6) | 3.1 (1.0) | 4.1 (0.6) | 3.4 (1.1) |
| I can communicate the quality of medical tests in an understandable way. | 2.8 (1.1) | 3.3 (1.1) | 4.5 (0.5) | 3.5 (1.1) | 3.9 (0.8) | 3.3 (1.1) |
| I can detect bias in study data. | 2.5 (0.9) | 2.8 (0.9) | 4.3 (0.7) | 3.3 (1.1) | 4.2 (0.8) | 3.3 (0.9) |

*Appendix Table 5. Situational judgment test scores at baseline, posttest and follow up*

| **Parameter** | **Baseline** | **n** | Δ (Posttest) | **n** | Δ (Follow up) | **n** |
| --- | --- | --- | --- | --- | --- | --- |
| **Total** |  |  |  |  |  |  |
| Intervention Group Mean (SD) | 66.22 (14.02) | 32 | 18.66 (12.06) | 29 | 19.12 (15.75) | 25 |
| Control Group Mean (SD) | 55.61 (15.06) | 31 | 0.27 (7.75) | 26 | 3.35 (12.94) | 23 |
| Difference Intervention and Control M (95% CI; p) | - | - | 18.39 (12.83-23.94; <0.001) |  | 15.77 (7.36-24.19; <0.001) |  |
| Cohen's d M (95% CI) | - | - | 1.79 (1.14-2.39) |  | 1.09 (0.47-1.68) |  |
| **Scenario 1 - Invitation to a meal by a pharmaceutical rep** | | | | | |  |
| Intervention Group Mean (SD) | 14.75 (4.0) | 32 | 3.03 (4.0) | 29 | 4.27 (4.7) | 25 |
| Control Group Mean (SD) | 12.16 (4.7) | 31 | -0.27 (3.1) | 26 | 0.75 (3.5) | 23 |
| Difference Intervention and Control M (95% CI; p) | - | - | 3.3 (1.4-5.2; 0.001) |  | 3.5 (1.1-5.9; 0.005) |  |
| **Scenario 2 - sponsored patient information brochures** | | | | | |  |
| Intervention Group Mean (SD) | 12.5 (4.2) | 32 | 4.86 (4.6) | 29 | 5.81 (4.9) | 25 |
| Control Group Mean (SD) | 11.2 (3.6) | 31 | -0.31 (2.9) | 26 | -0.13 (4.7) | 23 |
| Difference Intervention and Control M (95% CI; p) | - | - | 5.2 (3.1-7.3; <0.001) |  | 5.9 (3.2-8.7; <0.001) |  |
| **Scenario 3 - sponsored ultrasound machine** | | | |  |  |  |
| Intervention Group Mean (SD) | 12.4 (4.8) | 32 | 3.31 (4.6) | 29 | 2.11 (4.7) | 25 |
| Control Group Mean (SD) | 9.3 (4.5) | 31 | -0.23 (3.2) | 26 | 0.54 (3.7) | 23 |
| Difference Intervention and Control M (95% CI; p) | - | - | 3.54 (1.4-5.7; 0.002) |  | 1.6 (-.8-4.0; 0.196) |  |
| **Scenario 4 - management of conflicts of interest for a guideline committee** | | | | | |  |
| Intervention Group Mean (SD) | 13.9 (2.8) | 32 | 3.45 (3.7) | 29 | 2.04 (3.7) | 25 |
| Control Group Mean (SD) | 12.2 (4.3) | 31 | 1.0 (2.95) | 26 | 1.26 (3.6) | 23 |
| Difference Intervention and Control M (95% CI; p) | - | - | 2.4 (.64-4.3; 0.009) |  | .78 (-1.3-2.9; 0.461) |  |
| **Scenario 5 - offer of a drug sample** | | | | | |  |
| Intervention Group Mean (SD) | 12.7 (5.1) | 32 | 4.0 (4.7) | 29 | 4.58 (5.1) | 25 |
| Control Group Mean (SD) | 10.7 (5.1) | 31 | 0.077 (4.0) | 26 | 0.70 (5.1) | 23 |
| Difference Intervention and Control M (95% CI; p) | - | - | 3.9 (1.6-6.3; 0.002) |  | 3.9 (.9-6.8; 0.011) |  |

*Appendix Table 6. Participation in events as well as offers and acceptance of gifts in the intervention (int) and control (con) group at baseline and follow up.*

| **Item** | **Baseline** |  | **Follow up** |  |
| --- | --- | --- | --- | --- |
|  | **Int** | **Con** | **Int** | **Con** |
| Participation in a sponsored educational event at least once | 7 (21.9%) | 9 (29.0%) | 8 (25.0%) | 6 (19.4%) |
| Participation in a sponsored other event at least once | 1 (3.1% | 5 (16.1%) | 1 (3.1%) | 3 (9.7%) |
| Offer of a small non-educational gift at least once | 14 (43.8%) | 15 (48.4%) | 9 (28.1%) | 9 (29.0%) |
| Acceptance of a small non-educational gift at least once | 14 (43.8%) | 16 (51.6%) | 8 (25.0%) | 8 (25.8%) |
| Offer of a small educational gift at least once | 9 (28.1%) | 12 (38.7%) | 6 (18.8%) | 9 (29.0%) |
| Acceptance of a small educational gift at least once | 9 (28.1%) | 12 (38.7%) | 6 (18.8%) | 8 (25.8%) |
| Offer of a professional gift at least once | 2 (6.3%) | 2 (6.5%) | 3 (9.4%) | 0 |
| Acceptance of a professional gift at least once | 2 (6.3%) | 1 (3.2%) | 2 (6.3%) | 0 |
| Asked to attend a sponsored lunch at least once | 1 (3.1%) | 1 (3.2%) | 2 (6.3%) | 1 (3.2%) |
| Attended a sponsored lunch at least once | 1 (3.1%) | 1 (3.2%) | 1 (3.1%) | 1 (3.2%) |
| Offer of a gift passed on by a doctor at least once | 14 (43.8%) | 16 (51.6%) | 4 (12.5%) | 10 (32.3%) |
| Acceptance of a gift passed on by a doctor at least once | 14 (43.8%) | 16 (51.6%) | 4 (12.5%) | 9 (29.0%) |
| Total offers of gifts or events median (interquartile range) | 1 (0-2) | 3 (1-5) | 0 (0-3) | 1.5 (0-3) |
| Total acceptance of gifts or events median (interquartile range) | 1 (0-2) | 2 (1-5) | 0 (0-2) | 1.5 (0-3) |

*Appendix 1. Detailed description of Video-observed Structured Clinical Examination (VOSCE)*

The students received two case vignettes at each time point in which they were to advise a patient either on the decision between two treatment strategies or on the decision for or against a diagnostic test. The materials they received contained a short description of the case as well as basic information on the disorder in question and biased informational material about the treatment or diagnostic test. The informational material contained all data that was necessary to judge the efficacy and risk of the intervention or screening, but some of it was in fine print or obscured by emphasis on less meaningful data (such as relative change in risk). For the treatment cases, the informational material presented information on fictional drugs for chronic disorders (depression, multiple sclerosis and Crohn’s disease). In each case, the data showed that while one drug was more effective, the other had less risk of harms. However, the informational material emphasized the effectiveness of the one drug while minimizing its harms. For the screening cases (prostate cancer, colon cancer and breast cancer) data for real screenings were used but presented in a way that suggested that the screening had a larger effect than in reality.

The participants had one hour to prepare for each case. They were given a guideline designed to support their preparations by asking which data they thought especially relevant to the patient, how they would present it and what they needed to ask the patient to aid the decision. They were allowed to take these notes as well as the informational material into the conversation with the patient. Conversations with patients were timed to be no longer than 10 minutes. After 8 minutes, a warning sign was held up to prompt the participant to end the conversation. After 10 minutes, the camera was turned off and participants were informed that the time was over. No feedback was given.

Standardized patients were trained in the cases by the same trainer. For each case, there were several different actors (up to three). They were prompted to be inquisitive and decisive without asking for specific numbers; when they felt adequately informed, they were asked to make a decision, however, it was made clear that a decision could be deferred if they felt that the student had not informed them adequately.

*Appendix 2. Adaptation and scoring details of the rating scale*

The rating scale was adapted from Paul Han et al.(18). The original scale was first translated into German. Within the risk communication process part of the scale, one item was eliminated (“Demonstrated responsiveness, empathy, respect, professionalism”) because it was judged to be unclear as well as redundant with another item (“Showed empathy”). Another item (“Closed consultation appropriately”) was adapted to read “A decision was made” because this was deemed to be more easily defined and thus better ratable as well as more relevant to the risk communication process. Within the risk communication content part of the scale, one item was eliminated (“Placed the magnitude of risks in context by comparing to risks of other outcomes (e.g., other diseases, treatments, familiar events)”) because we did not judge it to be an important part of the risk communication content.

After adaptation of the scale, a detailed rating guideline was developed that gave clear definitions and examples for each of the possible ratings on the Likert-Scale from zero (not implemented) to three (implemented very well).

NDe and CK conducted test-ratings with the resulting scale using the videos from the initial pilot. In an iterative process, the rating-guideline was further revised to clarify certain definitions and the final guideline resulted in consistent ratings between NDe and CK. The final scale consists of 20 items, of which 12 rate the risk communication process (RCP), i.e. generic communication skills, and 8 rate the risk communication content (RCC), i.e. the key risk information being communicated. For each time point, scores for the screening and therapeutic intervention station were added, leading to a maximum possible of 120 on risk communication performance; 72 for risk communication process and 48 for risk communication content. Higher scores indicate better risk communication performance.

The final videos were rated by student aids after the participants had completed the assessments.

*Appendix 3. Situational judgment test – example*

Following, different scenarios are described that you may be confronted with during your medical education or your later medical practice. Afterward, different options of how you may react will be described. Please judge for each option how you would behave. Note that you are judging each option separately. Your answers may contradict each other.

1. You are doing a rotation on a general internal medicine ward. The resident who is supervising you is invited to a meal by a pharmaceutical representative. She asks the rep whether you as a future doctor may also come along and the representative invites you to dinner as well. How would you most likely behave?

|  | Never | Very unlikely | Unlikely | Likely | Very likely | Definitely |
| --- | --- | --- | --- | --- | --- | --- |
| 1. I accept the invitation because I can discuss new medication with the pharmaceutical representative and learn a lot. | □ | □ | □ | □ | □ | □ |
| 1. I accept the invitation because influence on me would not have any effect because I am not yet allowed to prescribe medication. | □ | □ | □ | □ | □ | □ |
| 1. I don’t accept the invitation because I want to remain independent. A meal with a pharmaceutical representative could influence me unconsciously. | □ | □ | □ | □ | □ | □ |
| 1. I accept the invitation because I know that the pharmaceutical representative is trying to influence me as a future doctor, so I am immune to the influence. | □ | □ | □ | □ | □ | □ |
| 1. I accept the invitation because I want to reward myself for the extreme effort that goes into my studies. | □ | □ | □ | □ | □ | □ |

*Appendix 4. Situational Judgment Test – Scoring*

The SJT consisted of five scenarios with five behavioral options each. Students were asked to rate the behavioral options by how likely it was that they would behave this way in the described situation on a 6 pt. Likert scale (from never to definitely). The scenarios were explicitly chosen to cover different types of conflicts of interest and different aspects of how to manage them. For each scenario, the desirable way to behave was determined by asking experts, i.e. in the example SJT of an invitation by a pharmaceutical representative, it would have been desirable not to accept the invitation. The reasons for choosing the respective behavior were not rated. The students could gain 0 to 5 points on each item. For items describing a desirable behavior, they gained more point the more likely they thought it was that they would show this behavior; for the other items, the scale was reversed. For the final score, the scores for each item were added up to result in a score of up to 125 points.

In addition, for each SJT, two options were chosen that described the worst possible way to behave and the best possible way to behave, respectively. Scores for these two items per scenario were also added up to result in a maximum score of 50 points.
